# Cancer Cell Sensitivity to Redox-Cycling Quinones is Influenced by NAD(P)H: Quinone Oxidoreductase 1 Polymorphism

**DOI:** 10.3390/antiox8090369

**Published:** 2019-09-02

**Authors:** Christophe Glorieux, Pedro Buc Calderon

**Affiliations:** 1 Metabolism and Nutrition Research Group, Louvain Drug Research Institute, Université catholique de Louvain, 1200 Brussels, Belgium; 2 Facultad de Ciencias de la Salud, Universidad Arturo Prat, 1110939 Iquique, Chile

**Keywords:** NQO1, NQO1*2, polymorphism, quinone, breast cancer, menadione, lapachone, doxorubicin, ascorbate, oxidative stress

## Abstract

Background: Cancer cell sensitivity to drugs may be associated with disturbed antioxidant enzymes expression. We investigated mechanisms of resistance by using oxidative stress-resistant MCF-7 breast cancer cells (Resox cells). Since nicotinamide adenine dinucleotide phosphate (NAD(P)H): quinone oxidoreductase-1 (NQO1) is modified in tumors and oxidative stress-resistant cells, we studied its role in cells exposed to β-lapachone, menadione, and doxorubicin. Methods: Normal mammary epithelial 250MK, MCF-7, and Resox cells were employed. NQO1 expression and enzyme activity were determined by quantitative polymerase chain reaction (RT-PCR), immunoblotting, and biochemical assays. Dicoumarol and gene silencing (siRNA) were used to modulate NQO1 expression and to assess its potential drug-detoxifying role. MTT (3-(4,5-dimethylthia-zolyl-2)-2,5-diphenyltetrazolium bromide) or clonogenic assays were used to investigate cytotoxicity. NQO1 variants, NQO1*1 (wt), and NQO1*2 (C609T), were obtained by transfecting NQO1-null MDA-MB-231 cell line. Results: Resox cells have higher NQO1 expression than MCF-7 cells. In 250MK cells its expression was low but enzyme activity was higher suggesting a variant NQO1 form in MCF-7 cells. MCF-7 and Resox cells are heterozygous NQO1*1 (wt)/NQO1*2 (C609T). Both NQO1 polymorphism and NQO1 overexpression are main determinants for cell resistance during oxidative stress. NQO1 overexpression increases cell sensitivity to β-lapachone whereas NQO1*2 polymorphism triggers quinone-based chemotherapies-sensitivity. Conclusions: NQO1 influences cancer cells redox metabolism and their sensitivity to drugs. We suggest that determining *NQO1* polymorphism may be important when considering the use of quinone-based chemotherapeutic drugs.

## 1. Introduction

**Figure 1:**
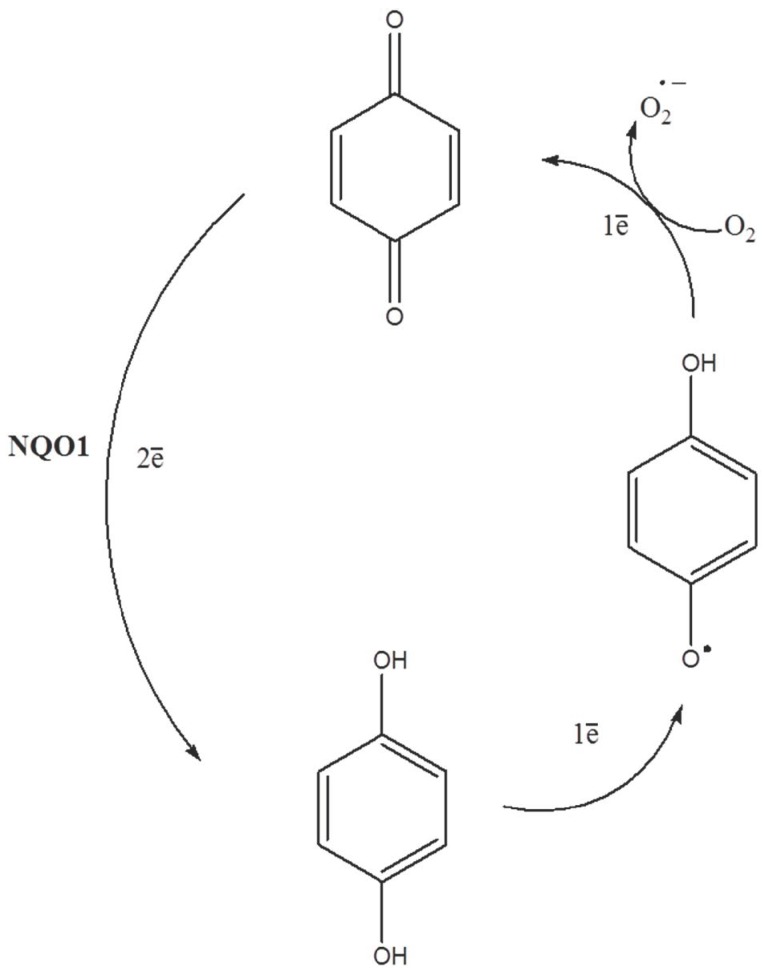
Quinone reduction by nicotinamide adenine dinucleotide phosphate (NAD(P)H): quinone oxidoreductase 1 (NQO1) and quinone redox cycling generating reactive oxygen species (ROS).

It has been generally accepted that nicotinamide adenine dinucleotide phosphate (NAD(P)H): quinone oxidoreductase 1 (NQO1, also known as DT-diaphorase) facilitates detoxification of quinone-based compounds by reducing their quinone nucleus [1,2,3]. Indeed, NQO1 through a two-electron reduction process, transforms quinone (Q) into a hydroquinone (QH2). In some occasions and depending on its stability, QH2 may be back oxidized to a semiquinone free radical (SQ•) and further to the original quinone while molecular oxygen is reduced to superoxide anion (Figure 1). In this context, since NQO1 is frequently overexpressed in a variety of tumors [4,5,6], the use of bioactive quinones has been exploited therapeutically because they are activated by NQO1 [7,8].

A different NQO1 expression pattern, at both protein levels and enzyme activity, has been found in tumors and normal tissues [9]. Recently, we found that some antioxidant enzymes, including NQO1, were overexpressed in MCF-7 breast cancer cells chronically exposed to hydrogen peroxide (H_2_O_2_) and by consequence becoming resistant against such oxidative stress, the so-called Resox cells [10]. Moreover, we detected a genomic gain of the chromosomal band 16q22 (where *NQO1* gene is located) in Resox cells as compared to parental MCF-7 cells suggesting an amplification of *NQO1* gene [11]. 

In addition, the existence of a polymorphism has also been noted. Indeed, two single nucleotide mutations have been reported: The C609T polymorphism, corresponding to a Pro187Ser change in the enzyme and described as NQO1*2, and C465T polymorphism, corresponding to an Arg139Trp change in the enzyme and described as NQO1*3. These polymorphisms are associated with a decreased enzyme activity [12,13]. Here, we paid particular attention to the study of *NQO1* polymorphism, using a model of NQO1-null MDA-MB-231 cells stably transfected with either NQO1*1, the wild-type form of *NQO1*, or the NQO1*2 polymorphism. Depending on both the genotype and the chemotherapeutic drug, the final antitumor outcome can be dramatically influenced by NQO1 activity. The aim of the study was to investigate in such experimental model the role of NQO1 polymorphism on cancer cell sensitivity to quinone-based therapeutic drugs. 

## 2. Materials and Methods

### 2.1. Cell Lines and Culture Conditions

MCF-7 cells, a human breast derived cell line, was obtained from ATCC (Manassas, VA, USA). By exposing them to chronic oxidative stress, they acquired resistance against a pro-oxidant treatment; therefore, they were named Resox cells [10]. MDA-MB-231 cells (ATCC) were kindly offered by Dr. Akeila Bellahcene (Metastasis Research Laboratory, Giga Cancer, Liège, Belgium). DMEM medium containing 10% fetal calf serum (10%), penicillin (100 U/mL), and streptomycin (100 μg/mL), obtained from Gibco (Grand Island, NY, USA), was used for cell cultures. Dr. Martha Stampfer (Lawrence Berkeley National Laboratory, Berkeley, CA, USA) kindly provided 250MK cells, a human mammary epithelial cell line. They were maintained in a special medium (M87A + CT + X) and further used between eight and 10 passages [14]. Cell cultures were kept at 37 °C under an atmosphere of 95% air/5% CO_2_ and 100% humidity. Dicoumarol, sodium L-ascorbate, menadione sodium bisulfite, β-lapachone, and doxorubicin hydrochloride were purchased from Sigma (St Louis, MO, USA). 

### 2.2. Stable Transfection

pKK233-2 plasmids containing human NQO1*1 (wild-type) and NQO1*2 (C609T) cDNA (NCBI Reference Sequence: NM_000903.3) were a kind gift of Dr. David Ross [15]. The following primers were used to amplify by PCR the different cDNAs. Forward 5′-ccgaagcttgccatggtcggcagaagagc-3′ and Reverse 5′-ccgggtacctcattttctagctttgatct-3′ (Sigma, St Louis, MO, USA). HindIII and KpnI (Fermentas, Vilnius, Lithuania) were used as restriction enzymes and insert DNA were then cloned into pcDNA3.1 plasmid from Invitrogen (Grand Island, NY, USA). The transfection of MDA-MB-231 cells were done with different plasmids (1 μg), followed by four week-selection of exposure to 1 mg/mL neomycin (Invivogen, San Diego, CA, USA). Both NQO1 enzyme activity and protein levels were used to characterize stable transfecting clones. Only clones with high NQO1 activity and similar NQO1 protein levels were chosen for further studies.

### 2.3. Small Interfering RNA Transfection Procedure

The transfection of cells with siRNA against NQO1 (ON-TARGET plus SMART pool siRNA) was done with Dharmafect reagent 1, according to Dharmacon protocols (Lafayette, CO, USA). The transfection technique was conducted for 24 h at 50% cell confluence, using 0.1 μmol/L siRNA solution. Transfected cells were utilized 48 h after such transfection procedure.

### 2.4. Western Blots Assay

Protein sample preparation, protein quantification, and western blot analyses were done as reported elsewhere [16]. Primary mouse antibodies were: β-actin (ab6276) from Abcam (Cambridge, UK) and NQO1 (sc-32793) from Santa Cruz Biotechnology (Santa Cruz, CA, USA). Protein bands were revealed by chemiluminescence, according to procedures given by the ECL detection kit (Pierce, Thermo Scientific, Rockford, IL, USA). ImageJ software (http://rsb. info.nih.gov/ij/) was used to quantify protein bands.

### 2.5. Measurement of NQO1 Enzyme Activity

The activity of NQO1 was measured following the reduction of cytochrome C in the presence of NADH (reduced nicotinamide adenine dinucleotide) as reported by Fitzsimmons et al. [17]. Briefly, 2 × 10^6^ cells were seeded in a 100 mm-culture dish containing 7 mL of incubation medium. Afterwards, when cells reached confluence, they were washed twice with ice-cold PBS (phosphate buffer saline) and further lysed with 1% Triton X-100 in PBS (500 μL), containing cocktails to inhibit both proteases (Sigma, St Louis, MO, USA) and phosphatases (Millipore, Merck KGaA, Darmstadt, Germany). Afterwards, samples were cold conserved for 5 min followed by sonication for 5 s at 4 °C using the Labsonic U sonicator (B Braun Biotech International, Melsungen, Germany). Each sample was prepared in duplicate, in the absence or in the presence of 10 μmol/L of dicoumarol (a well-known NQO1 inhibitor). The mixture (1 mL) contains 77 μmol/L of cytochrome C, 200 μmol/L NADH, 10 μmol/L menadione, 0.14% bovine serum albumin, and 50 mmol/L Tris-HCl pH 7.5. This mixture was incubated for 20 min at 37 °C and the tested sample (5 μL) was added. Cytochrome C reduction was read during 2 min at 550 nm and the calculated ΔOD/min, in the absence and in the presence of dicoumarol, was used to determine enzyme activity. The specific NQO1 activity was calculated by using a cytochrome C molar extinction coefficient of 21.1 mM/cm and results were expressed as nmol of cytochrome C reduced/minute/mg of protein. Intracellular protein levels were measured using a BCA (bicinchoninic acid) protein kit (Thermo Scientific, Rockford, IL, USA). All reagents were from Sigma (St Louis, MO, USA).

### 2.6. MTT Reduction Assay

The metabolic status of cells was evaluated by recording the formation of blue formazan crystals due to the reduction of MTT (3-(4,5-dimethylthia-zolyl-2)-2,5-diphenyltetrazolium bromide) by cellular dehydrogenases. In brief, 1 × 10^4^ cells/well were plated onto 96-well plates and, when confluence was achieved, cells were further exposed to the indicated treatments. Afterwards, cells were washed two-times using PBS and further incubated with MTT (0.5 mg/mL) for 2 h. The blue formazan crystals were solubilized with DMSO (100 μL/well) and the absorbance of blue-dyed solutions were read at a wavelength of 550 nm. The absorbance obtained by measuring untreated control cells was taken as 100%. 

### 2.7. Proliferation Assay

The clonogenic assay was done as follows: about 500 cells were seeded in six-well plates at a single-cell density. After overnight incubation, cells were exposed for 24 h to the respective quinones. Furthermore, they were washed with warm PBS, a new fresh medium was added, and cells were allowed to proliferate for 10 days. Clonogenic survival was measured by fixing and staining colonies using crystal violet and further counting their number. The number of colonies calculated under control conditions was set as 100%.

### 2.8. Quantitative Polymerase Chain Reaction

The extraction of total cellular RNA was performed using TriPure reagent (Roche Applied Science Diagnostics, Mannheim, Germany). Reverse transcription was done using SuperScript II RNase H-reverse transcriptase and random hexamer primers were acquired from Invitrogen (Grand Island, NY, USA). For real-time PCR, the Sybr Green Supermix (Bio-Rad, Hercules, CA, USA) was used. Primer sequences were 5′-caaatcctggaaggatggaa-3′ (forward) and 5′-aagtgatggcccacagaaag-3′ (reverse) for human NQO1 (NCBI Reference Sequence: NM_000903.3); 5′-cttcactgctcaggtgat-3′ (forward) and 5′-gccgtgtggcaatccaat-3′ (reverse) for human EF1 (Elongation factor 1, as reference gene; NCBI Reference Sequence: NM_001402.6). Primers were designed by Sigma (St Louis, MO, USA). The incubation of samples was conducted for 5 min at 95 °C, and subsequently for 40 cycles of 10 s at 95 °C and 30 s at 60 °C followed by a melting curve. The fluorescence of samples was assessed after each cycle in a Bio-Rad IQ5 thermocycler. Results were calculated by means of the following equation: 2^−(Ct NQO1 − Ct EF1)^ and then compared to values obtained with untreated control cells.

### 2.9. Statistical Data Analyses

All experiments were done by at least three times. The experimental data were examined using either a one-way ANOVA or an unpaired two-tailed *t*-test, using GraphPad Prism software (GraphPad Software, San Diego, CA, USA). A value of *p* < 0.05 was set as level of significance.

## 3. Results 

### 3.1. NQO1 Expression and Activity in Non-Cancerous and Cancerous Breast Cell Lines.

**Figure 2:**
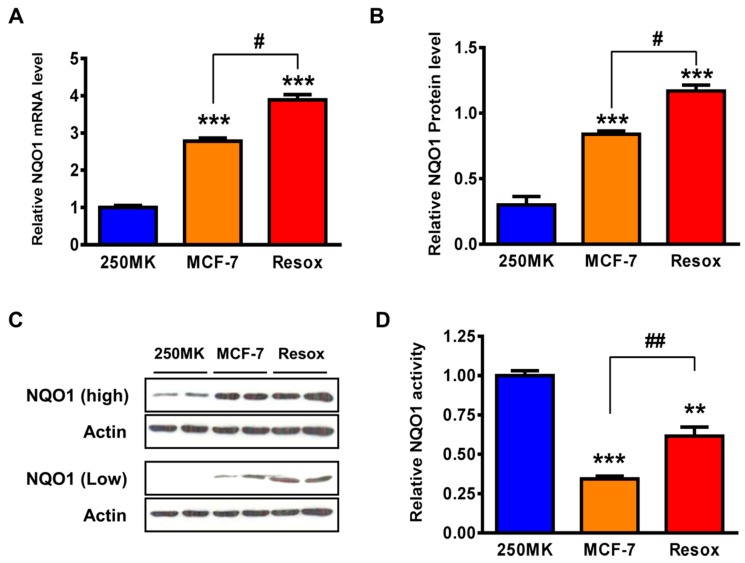
Basal levels of NQO1 expression in 250MK, MCF-7 and Resox cells. (**A**) NQO1 mRNA level was measured by real-time PCR. Data are means ± SEM (*n* = 3). (**B**) NQO1 protein levels normalized to β-actin were measured by immunoblotting. Data are means ± SEM (*n* = 3). (**C**) NQO1 expression detected by immunoblotting (low and high exposure times). (**D**) NQO1 enzyme activity. Data are means ± SEM (*n* = 3). Statistics: One-way ANOVA for (**A**,**B**,**D**). ** *p* < 0.01, *** *p* < 0.001 versus 250MK. # *p* < 0.05, ## *p* < 0.01 versus MCF-7. Abbreviations: NQO1: NAD(P)H: quinone oxidoreductase 1.

We first measured NQO1 expression in three cell lines: The normal human mammary epithelial 250MK, MCF-7 breast cancer, and Resox (MCF-7 resistant to an oxidative stress) cells. NQO1 was highly expressed in the breast cancer MCF-7 cells compared to 250MK cells (Figure 2A–C). Compared to parental MCF-7 cells, Resox cells have increased NQO1 mRNA (Figure 2A) and protein levels (Figure 2B,C). We then measured NQO1 enzyme activities and found those are also augmented in Resox compared to MCF-7 cells (Figure 2D). Surprisingly, NQO1 activity is enhanced in 250MK despite a low protein expression (Figure 2C,D). One explanation could be MCF-7 cells harbor a variant form of NQO1 and sequencing of the complete open reading frame (ORF) of the human *NQO1* gene in the three cell lines confirmed that 250MK cells were homozygous NQO1*1 (wild-type NQO1), whereas MCF-7 and Resox cells were heterozygous NQO1*1/NQO1*2 (data not shown). 

### 3.2. NQO1 Activity Correlates with Cancer Cell Sensitivity to Pro-oxidant Treatment.

**Figure 3:**
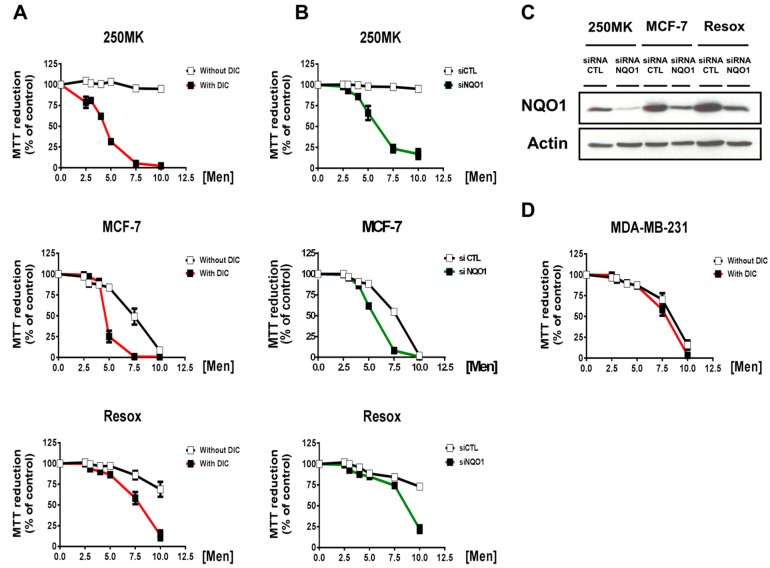
Sensitivity of mammary cells towards a pro-oxidant treatment. Cells were incubated for 24 h with various concentrations of ascorbate (Asc) ranging from 0 to 1 mmol/L, associated with menadione (Men) ranging from 0 to 10 μmol/L (ratio Asc/Men 100:1). (**A**) The specific NQO1 inhibitor dicoumarol (DIC) was tested at 25 μmol/L. Data are means (% of control) ± SEM (*n* = 3). (**B**) For genetic invalidation cells were transfected for 48 h with scrambled siRNA (siCTL) or specific siRNA against NQO1 mRNA (siNQO1). Cytotoxicity was evaluated using MTT assays. Data are means (% of control) ± SEM (*n* = 3). (**C**) NQO1 expression detected by immunoblotting. (**D**) MDA-MB-231 (pcDNA3.1) cells were treated with various concentrations of Asc/Men in presence or absence of 25 μmol/L dicoumarol. Cell viability was quantified by MTT assay. Abbreviations: DIC: dicoumarol; Men: menadione; NQO1: NAD(P)H: quinone oxidoreductase 1; si(RNA)CTL: control siRNA; si(RNA)NQO1: siRNA against NQO1.

Figure 3 shows that 250MK cells were resistant to the pro-oxidant treatment, in contrast to MCF-7 and Resox cells. Such oxidant treatment was induced by exposing cells to a H_2_O_2_-generating system (ascorbate and menadione), a mixture widely used in our laboratory to induce an oxidative stress [18,19,20,21,22]. Pharmacological inhibition of NQO1, by using dicoumarol, considerably increased the cytotoxicity in the three cell lines (Figure 3A). These results were then confirmed by genetic inactivation using a specific siRNA against NQO1 (Figure 3B,C). Unless ascorbate (Asc)/menadione (Men)-mediated cytotoxicity was not modified by dicoumarol in MDA-MB-231 cells (Figure 3D).

### 3.3. NQO1 Polymorphism Influences Cancer Cell Sensitivity to Redox-Cycling Quinones.

Because MCF-7 cells are heterozygous NQO1*1/NQO1*2 [12], we decided to study the importance of the *NQO1* polymorphism on the sensitivity of breast cancer cells to quinone-containing drugs. To this end, the wild-type form of *NQO1* (NQO1*1), or the variant form of the enzyme NQO1*2, were overexpressed in NQO1-null MDA-MB-231 cells. 

**Figure 4:**
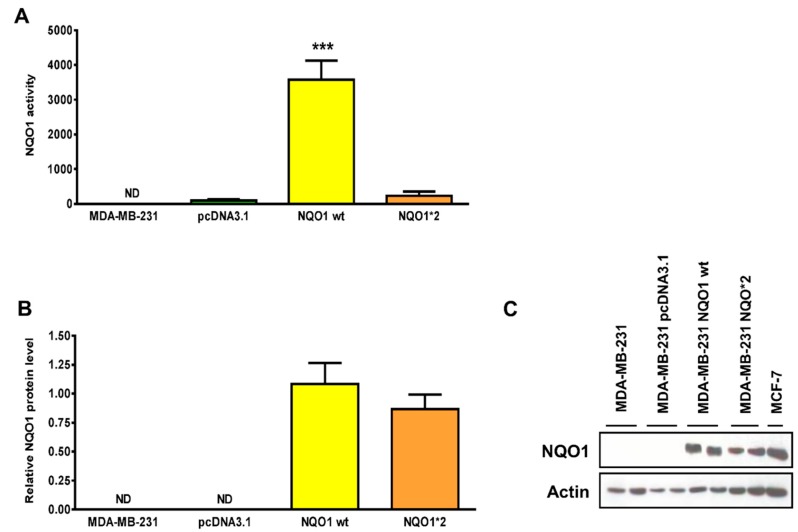
MDA-MB-231 cells overexpressing WT or C609T mutant forms of NQO1. (**A**) The specific NQO1 enzyme activity was calculated using a kinetic spectrophotometric method as explained in the respective experimental section. Results are expressed as nmol of cytochrome C reduced/minute/mg of protein. Data are means ± SEM (*n* = 3). *** *p* < 0.001 versus pcDNA3.1 (unpaired *t*-test) (**B**) NQO1 protein levels were quantified and normalized to β-actin. Data are means ± SEM (*n* = 3). (**C**) NQO1 expression was detected by immunoblotting. Abbreviations: NQO1: NAD(P)H: quinone oxidoreductase 1.

As shown in Figure 4A, NQO1*1-overexpressing cells had about a 10-fold greater NQO1 activity than NQO1*2-overexpressing cells, despite the fact that both cells have similar NQO1 protein levels (Figure 4B,C). This result confirms that the presence of the polymorphism NQO1*2 is associated with a decrease in NQO1 activity [13].

These newly generated cell lines were further exposed to menadione, doxorubicin, and β-lapachone. Using clonogenic survival assays (Table 1), we demonstrated that the expression of NQO1*1, the wild-type form with normal activity, was a main factor of cancer cell resistance vis-à-vis menadione and doxorubicin. In addition, the NQO1*1 expression was essentially linked to cell sensitivity toward β-lapachone. In contrast, expression of the NQO1*2 variant, which presented virtually no NQO1 activity, had no significant influence on cell survival, compared to MDA-MB-231 cells expressing the empty vector.

Similar findings were observed with MTT assay whereas inhibition of NQO1 with dicoumarol abolished the effect of the quinones (menadione and β-lapachone) on the cell viability (Table 2). Altogether these data suggest that, beyond the expression of NQO1 itself, the *NQO1* polymorphism has a major influence on the sensitivity of cancer cells to some chemotherapeutic drugs.

## 4. Discussion

To explore how NQO1 may affect both the activity and the detoxification of quinone-bearing compounds, we selected three different types of quinones displaying different physical-chemistry properties and molecular descriptors such as lipophilia and redox potential. Indeed, β-lapachone is an ortho-naphthoquinone derivative and menadione is bearing an 1,4-naphthoquinone scaffold while doxorubicin belongs to the anthraquinone antitumor class. Since NQO1 protein levels are often increased in tumors as compared to healthy tissues [4,5,6], this enzyme emerges as an attractive target for cancer therapy, because NQO1 bioactivates compounds like β-lapachone leading to a selective cancer cells toxicity [8,23,24,25].

A polymorphism has been described for the *NQO1* gene [12,13], resulting in the production of three variants: NQO1*1, the wild-type form; NQO1*2, a variant with a C609T substitution in exon 6; and NQO1*3, a variant with a C465T substitution in exon 4. Tumors with the NQO1*2 polymorphism usually have low NQO1 protein levels: For example, MDA-MB-231 cells, which are homozygous NQO1*2/NQO1*2, have very low NQO1 proteins and are nearly undetectable by immunoblotting; thereby they are considered as NQO1-null [12,26]. In contrast, MCF-7 cells, which are heterozygous NQO1*1/NQO1*2, have high NQO1 expression. Upon NQO1 inhibition, either by using a pharmacological inhibitor or by genetic inactivation, 250MK cells which were naturally resistant to a pro-oxidant treatment (Asc/Men), were dramatically sensitized against this oxidant insult most likely explained because they are NQO1*1 homozygous. In contrast, dicoumarol has no major impact on Asc/Men-mediated cytotoxicity in MDA-MB-231 cells.

Due to the critical importance of *NQO1* polymorphism, both NQO1*1 and NQO1*2 isoforms were stably overexpressed in NQO1-null MDA-MB-231 cells. The overexpression of wild-type NQO1 made cells more resistant to menadione than cells transfected with pcDNA3.1, the empty vector. In contrast, the overexpression of NQO1*2 did not increase cancer cell resistance against menadione, most probably due to the strongly decreased NQO1 enzyme activity showed by this variant isoform. Doxorubicin follows a similar pattern as menadione, although its mechanism of cytotoxicity is rather more complex. Indeed, doxorubicin toxicity is partially mediated through a redox-cycling reactive oxygen species (ROS) formation. However, a major mechanism is associated with DNA-intercalating effects [27]. Furthermore, we analyzed the impact of *NQO1* polymorphism on β-lapachone, which is bioactivated by the enzyme. MDA-MB-231 overexpressing NQO1 cells, with high NQO1 activity, were more sensitive to β-lapachone treatment and less sensitive to doxorubicin and menadione than empty vector or NQO1*2 overexpressing cells. 

## 5. Conclusions

As conclusion, we suggest that determining *NQO1* polymorphism may be important when considering the use of quinone-based chemotherapeutic drugs. Compounds such as β-lapachone will not be useful, for example, when cancer cells are homozygous for the NQO1*2 isoform, because these types of compounds cannot be bioactivated by the NQO1*2 variant. However, compounds such as doxorubicin (and menadione) may be used in these circumstances because these types of compounds are not detoxified by the NQO1*2 variant.

## Figures and Tables

**Table 1:** Sensitivity of MDA-MB-231 cells overexpressing WT or C609T (NQO1*2) mutant form of NQO1 to quinones (clonogenic assay).

Parameter	Cell Type	Menadione (60 μmol/L)	Doxorubicin (0.05 μmol/L)	β-Lapachone (1.5 μmol/L)
Clonogenic Assay (Survival fraction)	MDA-MB-231 (pcDNA3.1)	3.7 ± 3.2	34.3 ± 8.5	87.1 ± 21.8
	MDA-MB-231 (NQO1 WT)	23.8 ± 5.1 **	58.6 ± 0.9 **	41.0 ± 28.9
	MDA-MB-231 (NQO1*2)	5.5 ± 4.6	40.4 ± 0.6	98.8 ± 18.0

Clonogenic survival was determined as described in Section 2. Cells were transfected with either the empty vector pcDNA3.1, NQO1 wild-type or NQO1*2 and incubated with quinones for 24h. Data are means ± SEM from three separate experiments. ** *p* < 0.01 versus MDA-MB-231 pcDNA3.1 (one-way ANOVA). Abbreviations: NQO1: NAD(P)H: quinone oxidoreductase 1.

**Table 2:** Sensitivity of MDA-MB-231 cells overexpressing WT or C609T (NQO1*2) mutant form of NQO1 to quinones and dicoumarol (MTT assay).

Parameter	Cell Type	Menadione (60 μmol/L)	Menadione + DIC	β-Lapachone (1.5 μmol/L)	β-Lapachone + DIC
MTT Assay (% of control)	MDA-MB-231 (pcDNA3.1)	4.6 ± 3.7	0.6 ± 0.1	44.3 ± 3.5	43.9 ± 4.9
	MDA-MB-231 (NQO1 WT)	30.0 ± 8.6 **	0.5 ± 0.2	21.4 ± 9.4 *	53.3 ± 3.1
	MDA-MB-231 (NQO1*2)	7.3 ± 3.8	1.4 ± 0.3 **	46.6 ± 8.3	38.7 ± 5.5

Cells were transfected with either the empty vector pcDNA3.1, NQO1 wild-type or NQO1*2 and incubated with quinones (menadione or β-lapachone) and/or dicoumarol (25 μmol/L) for 24h. Cell viability was determined by MTT assay. Data are means ± SEM from three separate experiments. * *p* < 0.05; ** *p* < 0.01 versus MDA-MB-231 pcDNA3.1 (one-way ANOVA). Abbreviations: NQO1: NAD(P)H: quinone oxidoreductase 1; DIC: dicoumarol.
